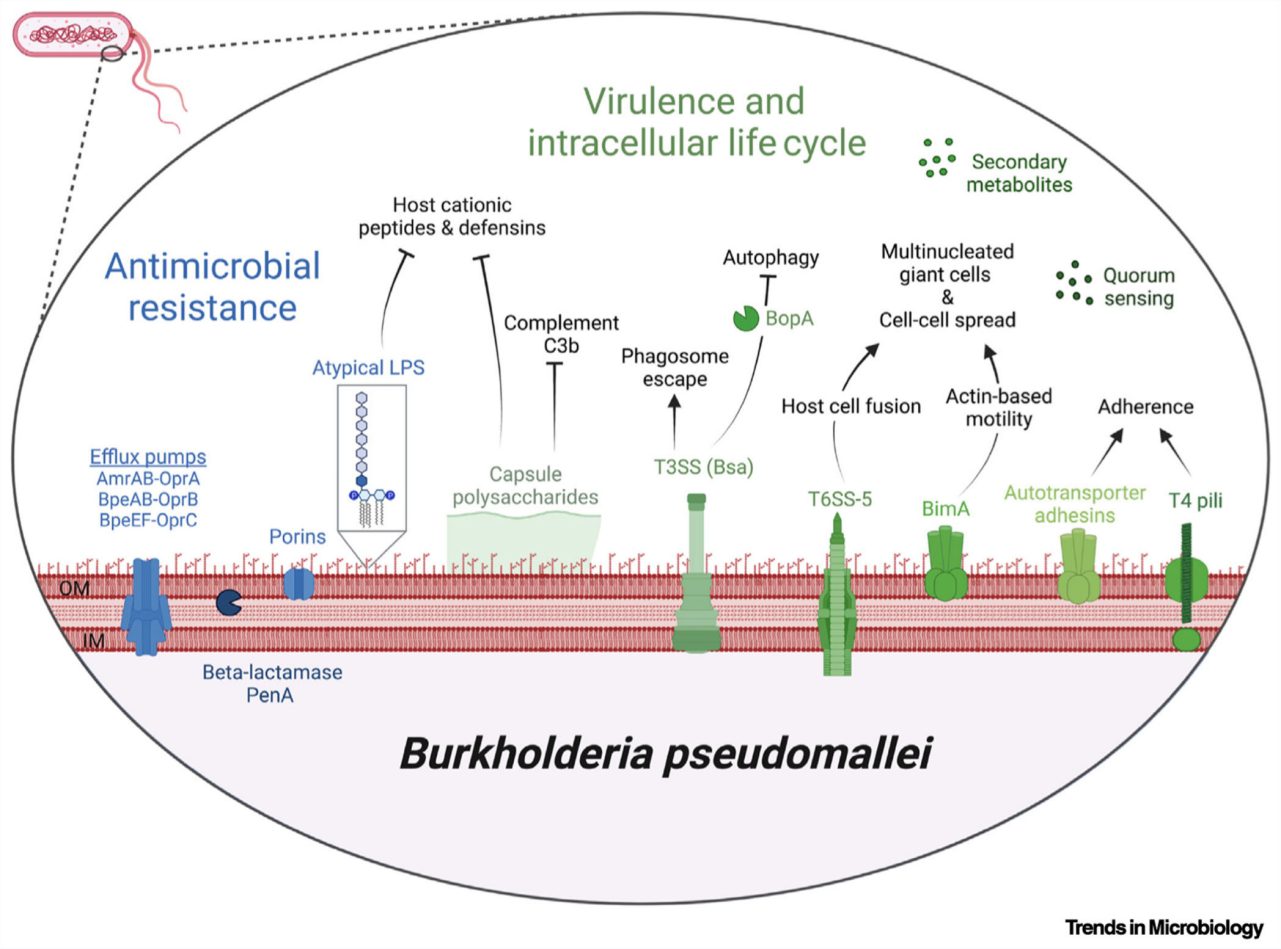# Burkholderia pseudomallei

**DOI:** 10.1016/j.tim.2023.07.008

**Published:** 2023-08-25

**Authors:** Erica D. Phillips, Erin C. Garcia

**Affiliations:** 1 University of Kentucky College of Medicine, Lexington, KY 40536, USA

**Keywords:** KINGDOM: Bacteria, PHYLUM: Pseudomonadota (Proteobacteria), CLASS: Betaproteobacteria, ORDER: Burkholderiales, FAMILY: Burkholderiaceae, GENUS: *Burkholderia*, SPECIES: *Burkholderia pseudomallei*

## Abstract

*Burkholderia pseudomallei* is a Gram negative, facultative intracellular bacterium that resides in the rhizosphere of tropical soils. *B. pseudomallei* causes melioidosis, which is transmitted by cutaneous entry, ingestion, or inhalation of contaminated soil or water. Infection with *B. pseudomallei* can cause a wide array of clinical symptoms such as pneumonia, bone, joint, skin, genitourinary, and central nervous system infections, as well as parotid abscesses in children. Mammalian virulence is linked to the *B. pseudomallei* intracellular life cycle, which begins with attachment and internalization by host cells. *B. pseudomallei* can infect a wide range of eukaryotic cells, including macrophages, monocytes, and neutrophils, as well as nonphagocytic cells. Once internalized, a type 3 secretion system (T3SS_Bsa_) facilitates *B. pseudomallei* escape from the phagosome, and the bacteria replicate in the cytoplasm. Autotransporter protein BimA mediates actin polymerization, enabling *B. pseudomallei* to spread, cell to cell, using actin-based motility. This process, coupled with the activity of a type 6 secretion system (T6SS-5), results in host membrane fusion and the formation of multinucleated giant cells. Capsule polysaccharides also contribute to virulence and evasion of host innate immunity. Treatment of *B. pseudomallei* infections is complicated by the organism’s intrinsic resistance to multiple classes of antimicrobials, largely due to an abundance of efflux pumps and reduced outer membrane permeability. While *B. pseudomallei* is commonly associated with endemic ‘hotspots’ in southeast Asia and northern Australia, there is increasing evidence that it is likely endemic in a large range of tropical and subtropical areas, including regions in Africa, South America, the Middle East, Central America, and the Caribbean. Soil and climate conditions favorable for *B. pseudomallei* survival are also found in additional areas worldwide. Consequently, it is important for clinical and public health laboratories located outside of high-endemicity areas to be aware of *B. pseudomallei*, as well as for improved diagnostic and reporting methods.

## KEY FACTS:

*B. pseudomallei* was first reported by Whitmore in 1911 as the causative agent of a ‘glanders-like’ disease.

Local regulatory guidelines for *B. pseudomallei* vary across the globe. The US Centers for Disease Control and Prevention categorizes the organism as a biosafety level 3 (BSL-3) pathogen and select agent.

Two chromosomes (~4 Mbp and ~3 Mbp) make up the large *B. pseudomallei* genome, which contains numerous genomic islands.

Environmental factors, such as soil type, temperature, moisture, pH, salinity, and nutrient levels influence *B. pseudomallei* prevalence in the soil.

Virulence mechanisms include capsule production, biofilm formation, adherence to host cells, resistance to reactive oxygen species, secondary metabolite production, quorum sensing, and intracellular survival mediated by flagellar- and actin-based motility, type 3 secretion, and type 6 secretion.

Colony morphology variants (including small-colony variants) differ in gene expression, virulence properties, biofilm production, and susceptibility to antibiotics.

*B. pseudomallei* is intrinsically resistant to multiple classes of antibiotics, including aminoglycosides, rifamycins, penicillins, cephalosporins, and cationic peptides.

*B. pseudomallei* is metabolically flexible, utilizing numerous carbon sources and encoding the potential to produce a large array of secondary metabolites.

## DISEASE FACTS:

Comprehensive global modeling estimated that there were 165 000 melioidosis cases in 2015, resulting in 89 000 deaths worldwide (estimated 54% mortality rate).

Diabetes mellitus is the leading risk factor for melioidosis. Other risk factors include chronic kidney or lung disease, liver disease, and heavy alcohol use, but an estimated ~20% of adult infections occur in patients who do not have defined risk factors.

Human-to-human transmission is rare, and infections are primarily due to environmental exposure to contaminated soil or water.

Melioidosis cases are seasonal and increase during the rainy season.

Typical incubation periods are 1–21 days, although decades-long latency periods following exposure have been reported.

Diagnosis is made through culture of patient samples (blood, sputum, urine, pus, cerebrospinal fluid), but misidentification of the organism may occur, particularly in non-endemic areas.

The initial phase of disease is treated intensively using intravenous ceftazidime or carbapenem over 10–14 days. Oral trimethoprim–sulfamethoxazole is administered during the subsequent eradication phase for at least 3 months and is also used prophylactically.

Although vaccine development is an active area of research, currently no vaccines are available to prevent *B. pseudomallei* infection.

## Figures and Tables

**Figure:**
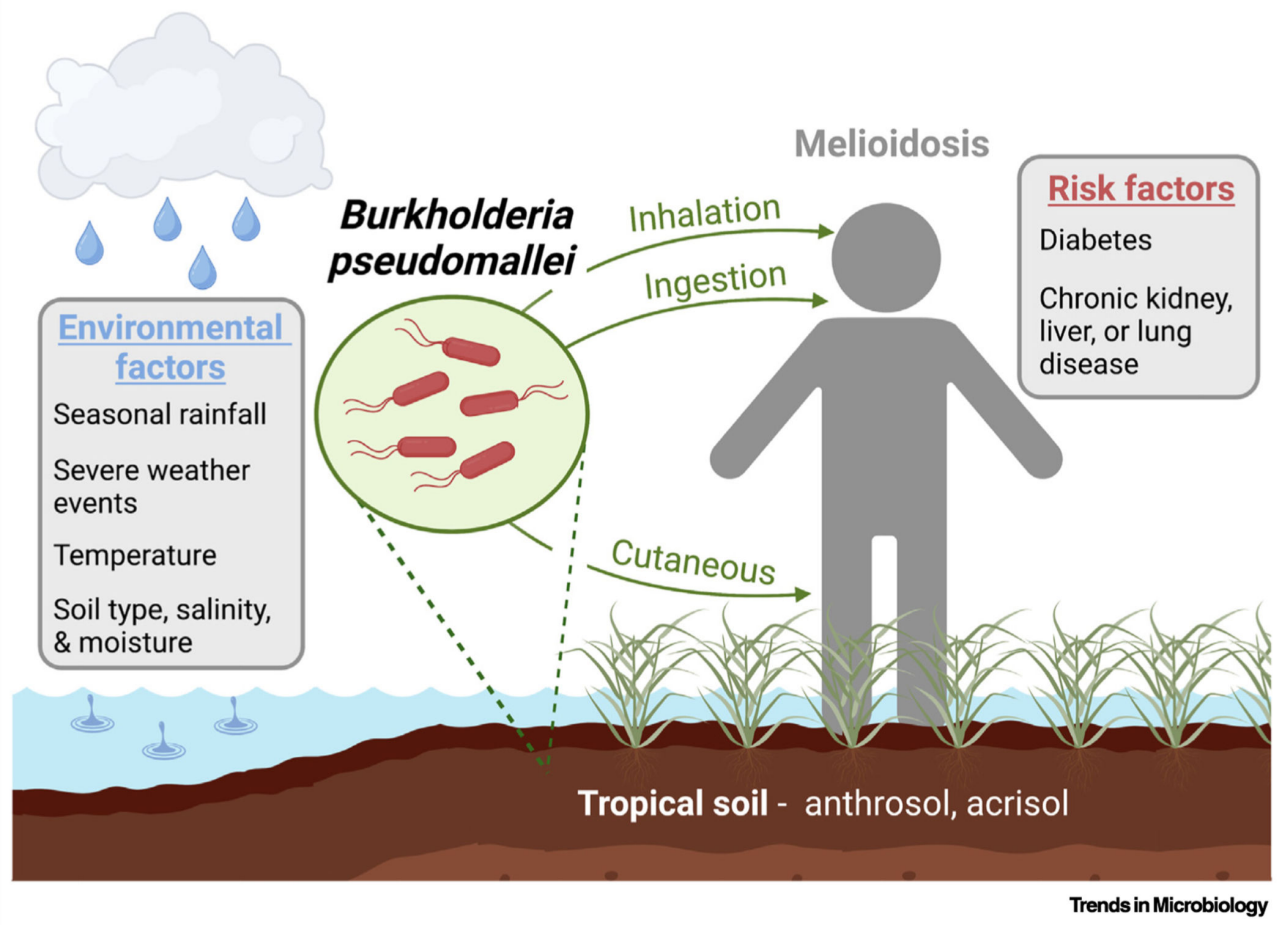


**Figure:**